# Adolescents’ use of music for pain management

**DOI:** 10.3389/fnhum.2025.1579130

**Published:** 2025-07-23

**Authors:** Rebecca J. Lepping, Lora L. Black, Kymberly A. Kline, Deanna Hanson-Abromeit, Andrea L. Chadwick, Dustin P. Wallace, William R. Black

**Affiliations:** ^1^Department of Neurology, University of Kansas Medical Center, Kansas City, KS, United States; ^2^Hoglund Biomedical Imaging Center, University of Kansas Medical Center, Kansas City, KS, United States; ^3^Department of Psychology, University of Kansas, Lawrence, KS, United States; ^4^Department of Psychiatry and Behavioral Health, The Ohio State University Wexner Medical Center, Upper Arlington, OH, United States; ^5^Department of Music Education and Music Therapy, School of Music, University of Kansas, Lawrence, KS, United States; ^6^Department of Anesthesiology, Pain, and Perioperative Medicine, University of Kansas Medical Center, Kansas City, KS, United States; ^7^Department of Pediatrics, University of Missouri-Kansas City School of Medicine, Kansas City, MO, United States; ^8^Pain Management Clinic, Children’s Mercy Hospital, Kansas City, MO, United States; ^9^Department of Pediatrics, Center for Biobehavioral Health, The Ohio State University College of Medicine, Columbus, OH, United States; ^10^The Abigail Wexner Research Institute, Nationwide Children’s Hospital, Columbus, OH, United States

**Keywords:** pediatric, pain, intensive interdisciplinary pain treatment, music, pain management strategies

## Abstract

To investigate the experiences of adolescents with chronic pain who participated in an intensive interdisciplinary pain treatment program, this secondary study analyzes the themes that emerged regarding the spontaneous utilization of music in coping strategies for chronic pain. During research interviews focused on coping skills and treatment engagement, participants spontaneously reported using music as an effective coping strategy for managing pain. A deductive thematic analysis revealed key themes related to their usage, including using music as a distractor, motivator and in other ways as coping strategies. Since participants indicated that music is essential to their experiences of coping with pain, incorporating these strategies could improve the effectiveness of treatment protocols. To this end, further investigation is necessary to assess the impact of music on adolescents with chronic pain, focusing on its role in enhancing interdisciplinary treatment.

## Introduction

Chronic pain, defined as pain that occurs for at least 3 months, impacts up to 15%–44% of adolescents aged 10–18 (de la Vega et al., 2018; Gobina et al., 2019). In this age range, the occurrence of chronic pain is associated with challenges in academic performance, increased school absences, and psychosocial difficulties including anxiety, depression, sleep concerns, and struggles with peer relationships (Logan et al., 2008; Simons et al., 2010; Hoffart and Wallace, 2014; Vervoort et al., 2014; Murray et al., 2020; Richardson et al., 2020). Adolescents with chronic pain are also at an increased risk for having chronic pain as an adult which can lead to additional healthcare costs and lower educational and/or vocational attainment as they age. As well, it is estimated that pediatric chronic pain costs approximately $19.5 billion annually in the United States (Groenewald et al., 2014). Thus, it is important that pediatric patients with chronic pain have access to appropriate treatment to help minimize the impact that these conditions have on their life and to promote optimal functioning.

For young people who are highly impaired by chronic pain, treatment may include Intensive Interdisciplinary Pain Treatment (IIPT) (Simons, 2013). IIPT programs typically include physical activity, desensitization, psychotherapy, education about pain mechanisms, learning and practicing stress management techniques, and an overall focus on moderation and normal functioning even when experiencing pain. As part of IIPT, patients are encouraged to develop and regularly use psychological, relaxation-based, and expressive coping skills to aid in the management of their conditions. These programs teach a variety of skills, allowing patients to identify which are most appealing and effective, increasing the likelihood of incorporating them into their daily lives after graduating from IIPT.

Music may be a particularly effective coping skill for adolescents with chronic pain (Scheufler et al., 2021). Music therapy has been utilized in multiple pediatric populations (Whitehead-Pleaux et al., 2006; Colwell et al., 2013; Millett and Gooding, 2018) and is associated with improved disease coping during their treatment, (Whitehead-Pleaux et al., 2006; Colwell et al., 2013) and overall quality of life (Scheufler et al., 2021). Music and music therapy programs may also be effective for pain coping in adolescent chronic pain and are included in some IIPTs. However, there is limited information about how children and adolescents with chronic pain use music outside of formal treatment programs.

Analgesia from music-based interventions (MBIs) may be due to psychological or physiological effects. Contextually, music unfolds over time in predictable ways leading to expectancies and providing context for what is coming next. This effect is amplified when the music is familiar, as it can evoke additional resources such as future thinking or enhancing sentimental feelings. Music listeners experience enhanced analgesic effects when they select their own music. Cognitive factors are also at play. Music can act as a cognitive distraction, redirecting attention away from painful feelings (Lunde et al., 2019). This phenomenon is not exclusive to music; similar effects can be achieved through other distraction methods, such as reading or listening to nature sounds (Villarreal et al., 2012). Emotion is a key component of the music experience as music has a compelling ability to trigger emotional responses (Juslin and Sloboda, 2001; Reybrouck and Eerola, 2017). For instance, emotionally positive music that is liked by the listener and has low arousal levels produces the strongest analgesic effects (Basinski et al., 2018). This has also been demonstrated to have clinical relevance as music listening interventions and music therapy have demonstrated effectiveness in alleviating anxiety and depression (Brandes et al., 2010; Aselton, 2012). Physiologically, the impact of music for pain may potentially be mediated through action on the parasympathetic nervous system, via vagal activation and lowering cardiac and respiration rates (Ribeiro et al., 2018). There have also been physiological effects observed in the brain, with music linked to the release of endogenous opioids and dopamine and activation of the descending pain modulatory system during the experience of pain (Blood and Zatorre, 2001; Salimpoor et al., 2011).

Given the multiple components of IIPT and the various strategies that are provided as part of these programs, we were interested in understanding how much adolescents who complete these programs go on to use music. To that end, we conducted a secondary analysis of interviews of adolescents who had completed a program to specifically examine uses of music in their responses to a general query about strategies that they are using to support their functional and health-related goals.

## Methods

The parent study interviewed a group of adolescents with Chronic Musculoskeletal Pain (CMSKP) who had completed an IIPT program at a large Midwestern children’s hospital about their resilience engaging in physical activity following the program. The IIPT program provides 8 h of treatment, 5 days a week, with treatment duration ranging from 4–6 weeks depending on each patient’s progress with specific functional goals. Treatment includes 4–5 h per day of physical and occupational therapy with a focus on exercise, desensitization, and a focus on normalizing physical functioning that had been impaired by pain. The other 3–4 h of each day includes individual and group psychotherapy (5 h per week), guided relaxation and meditation (2 h per week), yoga (2 h per week), art therapy (2 h per week) and music therapy (2 h per week), all provided by licensed or board-certified therapists. The IIPT program does not specifically focus on pain management, but instead focuses on improved functioning, returning to valued activities, and gaining confidence to adhere to recommendations following the program, which include daily exercise, desensitization, and proactive use of stress management strategies.

Initial analysis of the interview data found themes focused on the use of coping skills and engagement in recommended treatments for chronic pain that highlighted the role that music played in participants’ experience of chronic pain. Participation in music therapy, art therapy, and other relaxation techniques (yoga, meditation, etc.,) were provided as part of the structured program, and the interview question did not specifically ask about music. Thus, participants’ self-reports identified unsolicited thematic usage as to how they incorporated music in an unstructured manner with frequency that was notable and warranted further exploration into how participants utilized music and incorporated it into their experiences.

### Participants

Eligible participants were adolescents (ages 13–17 years) with a history of CMSKP who had completed an IIPT at the study’s institution and who had agreed to be contacted via email or recruitment flyers for future research. Ten participants enrolled in the study and completed the semi-structured interviews (*M*age = 16.45 years, *SD* = 1.35, range = 13.44–17.73). Ninety percent (*n* = 9) of the sample identified as female. Participants reported completing the program within the last 6 years (*M* = 2.23, *SD* = 1.70).

### Procedures

All study procedures were approved by Children’s Mercy Hospital Kansas City IRB and occurred remotely. Participants were recruited via email (i.e., existing patients that had agreed to be contacted for future research participation) or were referred by providers in pain management clinics. After providing written electronic parental consent and participant assent, participants completed surveys assessing background information and were provided additional information about the purpose and structure of the interview. Individual semi-structured interviews were conducted via an online HIPPA-compliant video communication platform (Microsoft Teams). Participants were informed that they could decline to answer any interview question or terminate the interview at any time.

### Semi-structured interviews

A trained psychology interviewer (JC), a female child clinical psychology graduate student, supervised by a male doctoral-level licensed psychologist/research faculty member (WB), conducted the individual interviews. The interviews, which ranged from 36–76 min in duration (*M* = 51.9; SD = 12.3) were audio recorded for transcription. The interviewer followed a semi-structured interview script constructed to elicit participants’ lived experiences with CMSKP and their engagement in physical activity, the recommended treatment from the IIPT, including use of helpful pain-focused management and coping skills. The interview covered topics such as general experiences with pain, effects of pain on participants’ lives, knowledge about pain and coping, resilience for managing and coping with pain, the role of resilience in general coping, and resilience for engagement in physical activity (Supplementary Data Sheet 1). Data collection continued until saturation was achieved for responses related to physical activity.

While the parent study was focused on the use of coping skills and engagement in physical activity for chronic pain and not on the use of music specifically, analysts of the parent study data observed that 7 out of the 10 participants discussed the role of music in their experience of chronic pain without prompting. To explore this observation more formally, we conducted a secondary qualitative analysis focused on these participants’ experiences with music, art, and relaxation techniques in relation to their chronic pain without a predetermined framework.

### Qualitative data analysis

To systematically examine the use of music reported by these participants, we conducted a qualitative thematic analysis of the physical activity focused interviews specifically for the use of music, the functions of music in that moment, and the situational appropriateness of that as a strategy. As music therapy and art therapy were offered in equal proportion during the program (1–2 h per week), we also examined the interview responses for uses of art.

Interview recordings were transcribed verbatim by a third-party transcription service and then checked for accuracy (JC). Participants were not involved in reviewing transcripts. Identifying information was removed prior to transcript review and coding. Interview transcripts were analyzed in Dedoose (version 9.0.17, Los Angeles, CA: SocioCultural Research Consultants, LLC) using deductive thematic analysis to identify, analyze, and report themes found within the data from a predefined theoretical framework (Braun and Clarke, 2006) used in the parent study. In the parent study, a pediatric pain resilience framework was used to develop the interview guide and inform deductive thematic analysis, highlighting various psychological factors that enable continued engagement in activities and behaviors while a person is experiencing pain (Goubert and Trompetter, 2017; Parsons et al., 2024). Two team members [JC and LB (a female doctoral-level licensed psychologist)] reviewed the transcripts and iteratively developed the initial codes, which were discussed and agreed upon by team members. All initial codes that discussed music were then analyzed separately by two team members (LB and WB) to be independently categorized into music-focused codes. Any discrepancies in coding were discussed until consensus was reached. In the event consensus was not reached, a third team member (RL) reviewed the responses and codes to decide on final coding categorization. All themes were developed from the iterative review of the seven participants who discussed music during their interview, where coders grouped similar responses together into codes which were then assigned a theme to best represent the group of responses. Social desirability bias (i.e., the tendency to present oneself and context in a more socially acceptable way that may not fully align with one’s reality) is often present in self-report research studies but may be especially important to acknowledge its role in qualitative research. While it is impossible to conduct qualitative interviews without the possibility of social desirability bias being present, we used several of the recommendations from Bergen and Labonte (2020) to limit such bias. These recommendations include indirect questioning, providing assurances, probing for additional information, requesting examples, and prefacing questions.

## Results

### Qualitative themes

*1. Using music as a distractor or to help pass the time.* Some of the participants reported that they used music to help distract them from the pain or when they were in overstimulating situations. Similarly, other participants described using music to help pass the time when engaging in other management techniques for their chronic pain.

*“But like if I’m in public of course, I’m usually – I’m just really big on distracters.* ***Music****, anything helps me focus on things*…*” (Participant 2, 17F)*

*“I*… *listen to* ***music*** *while going on runs. It just passes time.” (Participant 9, 15F)*

*“Doing something enjoyable while also doing something not so enjoyable. It makes it a little easier. So that would be one thing I’d recommend is just having something to do. So listening to* ***music*** … *having something else going on where it’s like I’m having some – this isn’t the enjoyment that I want, but it’s enjoyment while I’m doing something I need to do.” (Participant 3, 17F)*

*2. Using music as a motivator.* Participants also discussed how they would listen to music to help motivate them to keep using pain management recommendations, especially difficult ones such as physical activity. Of note, some also discussed the impact that music can have on both their mood and motivation.

*“So even when walking alone, I have a* ***playlist*** *that’s just full of super upbeat, cheery* ***music*** *with a beat that will just keep me going the entire time. I definitely don’t play slow* ***songs*** *or anything that you just. But just having that good* ***playlist****.” (Participant 2, 17F)*

*“I remember sometimes you guys would let us listen to* ***music*** *while we biked or elliptical or whatever that was, naturally boosted me. I remember that, like, I listened to some* ***motivational music****, for me, it was Fight Song by Rachel Platten, that was my fight song, like, I’m going to get through this, this is my fight song. Yeah, that was my fight song. And so, I was able to*… *go ahead and listen to some music, and that truly boosted my mood in order to get the work done.” (Participant 6, 15F)*

*“I like [engaging in physical activity] by myself and blasting* ***music***. *I find is what really just gets me on the zone.” (Participant 7, 15F)*

*3. Using music in other ways as a coping mechanism.* Other participants discussed how they incorporated music into their repertoire of coping mechanisms aside from just listening to it, such as analyzing lyrics or playing an instrument.

*“So a few days after I graduated from the program I experienced a really bad pain flaring*…. *And I was super upset about it*… *but with some coping strategies I’ve learned from the [IIPT] program, I picked myself up, stop feeling sorry for myself, said, okay, they gave me these resources need to start using them.*… *one of the things I loved was with [the music therapist] when we* ***listen to music*** *and*…*we just looked into the* ***lyrics****, it’s a whole new meaning.” (Participant 7, 15F)*

*“we decided I just could not do yoga or meditate, we decided that was just not in the picture. Yeah. And so, we had to come up with different ways that I could do that were reasonable. So, I’d listen to* ***music*** *or* ***play the piano*** *or write.” (Participant 1, 17F)*

*4. Art for relaxation, distraction, or in combination with music.* Four participants who had described their use of music also described using art, including drawing, painting, or needlework alone or in combination with music as coping mechanisms.

*“I really don’t know because I didn’t do physical activity, you know just by myself because I didn’t do anything. I’d rather sit at home and* ***draw*** *or something.” (Participant 1, 17F)*

*“wool needle felting [to help me] settle with the pain” (Participant 3, 17F)*

*“the one I use all the time is* ***music or drawing****.” “Just distracting my mind is what helps me the most. So, if I just think about something else or really focus on something else, like, for example* ***art****, yeah, it really helps.”(Participant 5, 16F)*

*“I think one of my main coping mechanisms before I came to your program and you gave me much better ones was definitely a lot of reading, a lot of sitting down, a lot of – I even got used to* ***painting and drawing*** *at that time. Like, you know, I never did that before, but when I was doing that, when I was, like, not even being able to move, I think like, just like, you know, either reading, drawing, stuff like that.” (Participant 6, 15F)*

## Discussion

During a semi-structured follow-up interview focusing on participants’ experiences with CMSKP, participants who had previously completed an IIPT program mentioned music as a resource for managing pain. Considering the options available in the IIPT and that participants are encouraged to utilize what is most effective and appealing for them, we took interest in strategies related to music usage. These were spontaneous responses about music and art to a general question about coping strategies for pain. Participants discussed the role of music in their experience with chronic pain and used it as a strategy. Themes that emerged from the participants use of music varied including distraction, motivation, coping mechanisms and psychological function of the music.

For participants who mentioned using music to distract themselves from the pain or to reduce social stress in social situations, this strategy aligns with existing literature indicating that music can serve as a cognitive distraction to shift attention away from discomfort. The capacity to overcome pain to participate in social contexts or overcome demands may have additional implications for adherence to coping strategies (Whitehead-Pleaux et al., 2006). The treatment often included a combination of active and passive coping strategies. For example, studies have shown that for those engaged in recommended exercise strategies, music can be used as a distraction from the physical symptoms they are having, while coping to help them with the emotional part of the pain experience (Saarikallio et al., 2019). In this way, listening to music can be a perceived as a way to control the external situation by managing inner/outer spaces, avoiding stressors from the environment, and self-regulating. Additionally, it is not always feasible to engage in relaxation strategies like yoga or deep breathing, so having a mix of skills or tools available that can best fit the situation can be helpful. Other participants used music as a distraction from the passage of time during over stimulating environments. Current research has highlighted similar use of music; the predictability of how music enfolds, especially within familiar music selections can tap into resources for future thinking, enhance sentimental feelings, serving as a distraction from the present moment and shifting away from the passage of time (Lunde et al., 2019). It has been shown that when individuals feel they have little control over their environment, music becomes an important tool for managing their internal mood to cope with the lack of control from the external world (Saarikallio et al., 2019). In this way, music may function as a mindfulness technique to bring engagement into the present moment and away from the focus on pain.

Participants also highlighted how they use music to motivate themselves to “push past the pain.” Music appears to be especially valuable for individuals who are either developing their sense of agency, such as youth, or facing a decrease in their ability to control actions and environment due to illness or difficult personal circumstances (Magee, 2017). By harnessing its emotional power, music can motivate individuals into actions (Krueger, 2014). For some, solitary listening can be considered a significant aspect of youth development, as the solitary experience fosters independence, serves as self-discovery, and personal mood regulation (Larson, 1995). For example, one participant shared how their playlist of music that was upbeat provided motivation to keep going. Creating personalized playlists further individualizes the experience of music listening by enhancing the effectiveness of a coping strategy.

Music is accessible to adolescents through listening, composing and performing. Along with the cultural phenomenon that teens use music to form identity, there is societal approval for music to voice protest or express thoughts that may conflict with societal norms. Accessibility and social acceptability make music unique in its usability compared to other coping methods. Additionally, the portability of music makes it easier to incorporate into daily routines as a coping mechanism. Thus, incorporating music into routines can integrate it as a lifelong strategy for managing pain.

## Limitations

There are some limitations to consider while evaluating these results. First, this is a secondary analysis of interview data collected from a relatively small sample of participants. The interview focused mainly on physical activity – the primary component of the IIPT – and the sample was determined sufficient to reach data saturation for the parent study. As this is a secondary analysis, sample size determination was not conducted for information about music use. Nevertheless, the high proportion of respondents spontaneously discussing their use of music was intriguing and yielded sufficient data for a preliminary exploration of themes highlighting potential areas for further exploration. However, these data are insufficient for generalizability of the findings. Future research collecting a larger sample size may generate findings that more broadly reflect how adolescents with CMSKP are using music for coping with pain. Additionally, participants were not specifically asked about their music usage, nor were they asked to provide their experiences with music such as playing an instrument or taking music classes; therefore, the data collected only reflects instances where adolescents with CMSKP mentioned music spontaneously as it relates to their pain. Furthermore, the limited sample size prevented exploration of cultural variations in music use for pain. Individual differences in how cultural background may have influenced preferences for musical styles and varying beliefs about music’s effectiveness in providing pain relief and healing (Becker et al., 2025). These were not evaluated in the current study. This study may, therefore, not fully capture the extent to which they engage with music in their lives as a coping strategy. Future studies should capture musical experience and training or other ways patients engage with music, including cultural considerations as potentially important factors that could influence a person’s use of music as a coping strategy for pain.

## Conclusion

This secondary thematic analysis illustrated that youth who had participated in IIPT also use music to help manage their pain and support their use of physical activity and other strategies learned in IIPT. The emergent themes in this study illustrate how music was functioning for this cohort of adolescents for distraction, motivation, and coping strategies. Music therapy shows promise in decreasing anxiety and promoting relaxation for adolescents participating in an IIPT program (Scheufler et al., 2021). The present observation is suggestive that more research is needed to investigate the role of music in the lives of adolescents with CMSKP and to further explore how music can make other parts of the interdisciplinary treatment more tolerable or effective.

Future studies should delve deeper into how music and art could be included into pain treatment and management plans particularly with targeted interventions that are aligned with IIPT program goals. Understanding the underlying physical and neurological mechanisms of music and art for pain management is key for incorporating these tools into prescriptive and personalized medicine. While there are promising studies showing improved quality of life, emotional regulation, and potential for cognitive improvement, this is outside the scope of the present investigation. Future research using functional magnetic resonance imaging (fMRI) or electroencephalography (EEG) is warranted to deepen our understanding how music changes the brain response to pain in adolescents, and cognitive or psychological testing could be used to measure broader effects. Moreover, qualitative approaches could foster more comprehensive insights into the specific aspects of music that provide the most benefit in managing pain. Adolescents use music in their everyday lives. Strengthening our understanding of the unique, person specific responses to music and how it can be used to manage pain for adolescents could provide a coping strategy in alignment with individual preferences that is also easily accessible and socially acceptable.

## Data Availability

The data analyzed in this study is subject to the following licenses/restrictions: Data used in this study are available from the corresponding author upon reasonable request. Requests to access these datasets should be directed to William.Black@nationwidechildrens.org.
