# Supplementary material for: Adolescents’ use of music for pain management
Source: Front Hum Neurosci. 2025 Jul 23;19:1579130. doi: 10.3389/fnhum.2025.1579130 (PMC12325222; doi:10.3389/fnhum.2025.1579130)
Supplement: Supplementary file 1 [file Data_Sheet_1.docx]

**Interview Guide**

Facilitators of Physical Activity Engagement in Adolescent Chronic Musculoskeletal Pain

*[After informed consent]:* Thank you for agreeing to talk with us today. We are trying to better understand what helps you do the things you need to do to manage your chronic pain and what are some of the things that keep you from being able to manage your pain. We particularly want to hear how these things help you or keep you from sticking to parts of your treatment recommendations, like physical activity.

Our discussion today will take about and hour and a half to 2 hours, and we will have breaks as needed. There are about 45 other adolescents and teens that are also talking with and helping us.

We’d like you to help us learn about how patients deal with pain and how they stick to their treatment recommendations. Even though it seems that nurses and doctors are the experts and should know how and what to ask kids about pain, we still want to make sure that we understand how you think about pain, and what you think helps you or keeps you from doing things when you have pain. When it comes to your own feelings and experiences, you’re the expert, right? So at first, we’ll talk about some of your own experiences and stories of different times that you were able to deal with pain and times where it was harder to deal with. Whatever you can tell us—and I’ll help all of you along—may help make up new questions that nurses and doctors can ask their patients.

Because your words and ideas are so important, we are going to tape/audio-record the conversation. That way I can focus on listening to you rather than on taking notes. [(TBD-name) is going to make a few notes too]. We’ll go back and listen to the recording and take more notes and then destroy the recording. Since the notes won’t have your name, nobody will be able to tell who said them. Also, in order to protect your privacy, I won’t use your last name. While we’re talking, remember that you can just say your first name. You don’t need to tell anyone your last name during the discussion, but it is also okay if you decide to say your last name. For clarity, your answers could be linked back to a study ID number, and the audio from this group may be linked to your name. However, as we spoke about during the consent process, we are being careful to protect your information and keep things confidential, including keeping identifiable information in locked cabinets and on secure networks.

There are no right or wrong answers to the questions, we are really just interested in your own personal opinions. And remember, if there is ever a question you don’t feel comfortable answering, that’s perfectly fine. Also, if the group is talking about something that you don’t want to talk about, you do not have to participate in that part of the conversation. Do you have any other questions before we start? Do you mind if we start the audio-recorder?

Any questions before we start?

OK, I’m going to ask your parents to go wait for you at (give location) or go to (give location) to participate in their own discussion group with other parents [if applicable]. They’ll still be close by, so if you need to see them or talk to them, just let me know. [Allow time for parents to leave room].

Now I am going to start the recorder. When I press this blinking red light, it stays red and tells us we’re recording.

**START RECORDER WITH PRE-RECORDED INFORMATION.**

Before we begin our discussion today, I would like to remind you of a few things:

As the moderator of today’s discussion, I will be helping to move the discussion along by asking some questions of the group and inviting you to discuss your thoughts among yourselves. There are no right or wrong answers. We expect that you will have different points of view and we respect all comments, even when they are different from what others have said. These differences are important to us and will also help to make this discussion interesting. We want to make sure that we hear from all of you, so please speak up and share your ideas.

Let’s cover some group rules.

- One at a time-speak clearly and slowly so that everyone can hear
- No right or wrong answers
- We want to hear from everyone
- Honor confidentiality-what’s said here, stays here
- You don’t have to answer a question if you don’t want to
- You can stop participating at anytime
- Please put cell phones on silent/vibrate

Now, I would like to go around the group and give introductions. Please tell us your first name, something about yourself (for example, favorite activity, sport, movie, etc), as well as where you have pain[note: names, ages, conditions].

*[Notes to interviewer: Bullet points are optional probes. Select as appropriate. Utilize general probes such as “tell me more” or “what do you mean by that” as needed to expand the discussion.]*

**Warm-up and general pain questions**

1. **General information about pain experience:** Everyone experiences pain differently. When you each think about some the different kinds of pain you’ve experienced, what are some words or expressions you could use to tell others what it’s like?
   - How would you describe your experience with pain to someone who has never experienced it?
   - [Compare/contrast different perspectives and different kinds of pain providing examples as needed: *toothache, cramps, sadness/loss*]*.*
2. **Effects of Pain:** Not only do people experience pain different, pain affects them differently too!
   - Think of a recent time when you were feeling or experiencing a lot of pain. How did it help you or hurt you in living your life?
   - How did it help or hurt you in doing the things that are important to you?
   - What do you do differently when you have pain?
   - What do *others* do when you are in pain?
   - How does pain affect your mood?
   - What are some things you think or tell yourself when you are having pain?
3. **Knowledge about pain management and coping:** So far, it sounds like while there are similarities to how you experience pain and how pain affects your lives, there are also some differences. We would like to know a little bit more about what you know about pain and what you try and do about it?
   - What are some of the things you have been told about how pain works?
   - What have you been told/do you remember about how to manage your pain?
   - What kinds of things, if anything, have you tried to manage with pain?
   - What kinds of things, if any, help your pain?
   - What kinds of things, if any, make your pain worse?

**Resilience for pain management and pain coping**

1. **General resilience:** We often hear that there are ups and downs with how well people can deal with their pain, or “good days” and “bad days.” Managing your chronic pain may be easier when your pain is less, BUT harder when your pain is worse. Is this something that any of you have experienced? (*Open discussion*)
   - What things are you still able to do when your pain is worse?
   - What things are harder for you to do when your pain is worse?
   - Now, I want you to think about some times when your pain was worse, but you **still** were able to deal with your pain. Tell me about some of those times.
   - What kinds of things helped you deal with your pain –
     1. OPTIONAL IF LIMITED RESPONSE:[…*and this can be many different things, from things you tell yourself or think in your head, support from friends and family, and others.]*
   - (*Elicit as many factors as possible and expand on them as needed to identify what factors are important to teens in helping them to manage pain).*
2. **Specific resilience factors for overall coping:** In our discussions with other teens, some specific things came out that others have said either helped them or kept them from living their life to the fullest extent possible, even with pain. Some of these you all have noted too. I would like to spend a little but more time discussing what you think.
   - **Negative Motivation:** Tell me about a time when you had pain and did not engage in an activity because you “didn’t want to” or “didn’t feel like it.”
     1. What made it so you did not choose to engage in the activity?
   - **Positive Motivation:** Tell me about a time when you had pain and still engaged in an activity when you wanted to?
     1. What made it so that you were able to engage in the activity?
     2. Thinking about times when you chose not to engage in the activity versus this time, what was different? What made it easier to engage in this activity?
   - **Negative Affect:** Tell me about a time when you had pain and did not engage in an activity because you were upset or were in a bad mood?
   - **Positive Affect:** Tell me about a time when you had pain and did an activity because you were feeling good emotionally or in a good mood?
     1. Tell me more about how your mood affected your ability to do an activity while having pain?
   - **Lacking Self-Efficacy:** Tell me about a time when you had pain, and wanted to do an activity, but you felt like you were not capable doing the activity while having pain.
     1. What were some of the things that made you feel like you could not do the activity while having pain?
   - **Positive Self-Efficacy:** Now, tell me about a time when had pain, wanted to do an activity, and DID feel like you were capable of doing the activity while having pain
     1. What were some of the things that made you feel like you could do the activity while having pain?
   - **Self-Talk/Pain Acceptance:** Earlier we talked about some of the things you tell yourself that help you deal with your pain or make dealing with your pain worse.
     1. When was a time that you told yourself that having pain was “part of it”, “how it is”, “I have to live with it,” ok “its ok that I have pain?” Others have also talked about “coming to terms” with managing their pain.
   - **Psychological Flexibility/Values:** Something else we have heard is that while there are successes in being able to manage pain, there are times when pain seems to have a “mind of its own”. Despite this, some teens report “doing things anyways” (going out with friends, school, events) because it was “important to them.”
     1. Tell us what things are important to you? Sometimes we refer to these things as our values.
     2. What do you do whenever what is important to you is in conflict with your pain?
     3. What do you tell yourself or how do you change your thinking?

Thank all of you so much for your participation thus far. We have covered so much ground and these kinds of conversations are VERY important to helping us better understand how you all experience and manage your pain. Something that has come up today (*if true*) is **OR** Something that has come up in other groups is the use of physical activity or exercise in pain management. Becoming and staying physically active is an important part of managing your pain. **AND** we know that this can be a very hard thing to do when you have pain.

So far we have talked about your ability to manage your pain more broadly. We would like to revisit some of the things we asked about, but focus more specifically on physical activity.

**Resilience for physical activity/exercise**

1. **Physical Activity Barriers/Promoters:** Tell us about a time that doing physical activity with pain was hard.
   - What kinds of things made this hard for you? (Note: while having pain is certainly one reason, try to think about other things too [thoughts, feelings, emotions, fatigue])
   - Tell us about a time that doing physical activity was easy or less difficult.
   - What kind of things made this easier.
2. **Resilience for physical activity:** What kinds of things helped you to engage in physical activity when you had pain – and this can be many different things, from things you tell yourself or think in your head, support from friends and family, and others.
   - (*Elicit as many factors as possible and expand on them as needed to identify what factors are important to teens in helping them to manage pain).*
3. **Specific resilience factors for physical activity engagement:** Similar to earlier, we are interested to know if there are some specific things (supports, strategies, self-talk, attitudes, ways of thinking) that help you engage in physical activity when you have pain.
   - **Negative Motivation:** Tell me about a time when you did not do regular physical activity or exercise because you “didn’t want to” or “didn’t feel like it.”
   - **Positive Motivation:** Tell me about a time when you did regular physical activity or exercise even though you did not want to.
     1. Tell me more about why you did or did not do a physical activity?
   - **Negative Affect:** Tell me about a time when you did not do regular physical activity or exercise because you were upset or were in a bad mood?
     1. Tell me more about how your mood affected your engagement in regular physical activity or exercise?
   - **Positive Affect:** Tell me about a time when you did regular physical activity or exercise because you were feeling good emotionally or in a good mood?
     1. Tell me more about how your mood affected your physical activity or exercise?
   - **Lacking Self-Efficacy:** Tell me about a time that you felt like you were not capable of doing regular physical activity or exercise – this is different than not wanting to.
     1. What were some of the things that made you feel like you could not do physical activity.
   - **Positive Self-Efficacy:** Now, tell me about a time when you DID feel like you could do regular physical activity or exercise while you were in pain.
     1. What were some of the things that made you feel like you could do regular physical activity or exercise while being in pain?
   - **Self-Talk/Pain Acceptance:** Earlier we talked about some of the things you tell yourself that help you deal with your pain or make dealing with your pain worse.
     1. When was a time that you told yourself that doing physical activity or exercise with pain was “part of it”, “how it is”, “I have to live with it,” ok “its ok that I have pain?” Others have also talked about “coming to terms” with doing regular physical activity to manage their pain.
   - **Psychological Flexibility/Values:** Something else we have heard is that while there are successes in being able to do physical activity with pain, there are times when pain makes it difficult to do anything at all. Despite this, some teens report “doing things anyways” (going out with friends, school, events) because it was “important to them.” This includes doing physical activity or exercise.
     1. Tell us why doing physical activity or exercise is or is not important to you.
     2. What do you do when you want to do a physical activity or exercise but your pain is “telling you” [Making air quote!] not to?
     3. What do you tell yourself or how do you change your thinking to help you complete physical activity?

Thank all of you again so much for sharing your thoughts and experiences with us. **Is there anything else that we should have asked you about or anything else you’d like to tell us?**

[*Thank again and dismiss*]
